# Recognition of Sedentary Behavior by Machine Learning Analysis of Wearable Sensors during Activities of Daily Living for Telemedical Assessment of Cardiovascular Risk

**DOI:** 10.3390/s18103219

**Published:** 2018-09-24

**Authors:** Eliasz Kańtoch

**Affiliations:** AGH University of Science and Technology, Faculty of Electrical Engineering, Automatics, Computer Science and Biomedical Engineering, Department of Biocybernetics and Biomedical Engineering, 30 Mickiewicz Ave. 30 30-059 Kraków, Poland; kantoch@agh.edu.pl

**Keywords:** wearable computing, smart clothing, activity recognition, telemedicine, sedentary behavior recognition, pervasive computing

## Abstract

With the recent advancement in wearable computing, sensor technologies, and data processing approaches, it is possible to develop smart clothing that integrates sensors into garments. The main objective of this study was to develop the method of automatic recognition of sedentary behavior related to cardiovascular risk based on quantitative measurement of physical activity. The solution is based on the designed prototype of the smart shirt equipped with a processor, wearable sensors, power supply and telemedical interface. The data derived from wearable sensors were used to create feature vector that consisted of the estimation of the user-specific relative intensity and the variance of filtered accelerometer data. The method was validated using an experimental protocol which was designed to be safe for the elderly and was based on clinically validated short physical performance battery (SPPB) test tasks. To obtain the recognition model six classifiers were examined and compared including Linear Discriminant Analysis, Support Vector Machines, K-Nearest Neighbors, Naive Bayes, Binary Decision Trees and Artificial Neural Networks. The classification models were able to identify the sedentary behavior with an accuracy of 95.00% ± 2.11%. Experimental results suggested that high accuracy can be obtained by estimating sedentary behavior pattern using the smart shirt and machine learning approach. The main advantage of the developed method to continuously monitor patient activities in a free-living environment and could potentially be used for early detection of increased cardiovascular risk.

## 1. Introduction

Demographic changes and increasing societal demands require more effective methods of providing health services based on novel technologies information and telecommunications (ICT), artificial intelligence and telemedicine. According to the Eurostat during the period from 2016 to 2080 older persons will likely account for an increasing share of the general population. Those aged 65 years or over will represent 29.1% of the population of EU Member States by 2080, compared to 19.2% in 2016. As a result, the EU Member States’s old-age dependency ratio is projected to almost double from 29.3% in 2016 to 52.3% by 2080. Therefore, this ratio will decrease from approximately three working-age people for every person aged 65 or over to one [1]. According to the Center for Disease Control and Prevention, the leading major causes of morbidity, disability, and mortality in 2015 were cardiovascular diseases (CVD) [2]. Treatment of these diseases is challenging for the healthcare systems in all developed countries due to the ageing population, limited access to the healthcare service and increasing healthcare costs. The main risk factors linked to cardiovascular disease are physical inactivity, poor diet, smoking and excessive alcohol consumption [3]. According to the recommendation of the European Guidelines on prevention of cardiovascular disease in clinical practice, the measures should be considered to promote a healthy lifestyle at the population level. Research revealed that the elimination of health risk behaviors would prevent at least 80% of CVDs [2]. According to the guidelines of the National Institute for Health and Care Excellence, the evidence on how to solve the problem of a sedentary lifestyle is not well developed and remains an area for further study [3].

Sedentary behavior analysis has been explored using wrists, hips or thigh mounted accelerometers using elastic belts. Sasai [4] overview of assessment of sedentary behavior using wearable devices and concluded that most commonly used wearable devices were activPAL, ActiGraph, and Active style Pro. However, those devices are not integrated with clothes but rather attached to the body using elastic belts. He noticed that those devices produce different outputs what makes it difficult to compare them. He found that the major disadvantage of wearable devices used for sedentary behavior analysis is the inability to detect the contextual information as well as time-consuming computation. Peterson et al. [5] showed that overall accuracy in measuring sedentary behavior using AcitGraph GT3X+ among university student was 94.7–97.8%. Koster et al. [6] examined the impact of accelerometer wear location on physical activity and sedentary behavior assessment among older adults. Participants were asked to simultaneously wear ActiGraph accelerometers on the dominant, and nondominant wrist and on the hip. Authors showed that hip and wrist-worn ActiGraph accelerometers might be used to recognize sedentary time with a moderate to high accuracy. However, Koster et al. [6] pointed out that wrist accelerometers underestimated the sedentary time and in some scenarios sitting time may not be distinguished from standing time-based on accelerometer data. A recent study by Trost et al. [7] using ActiGraph accelerometer on the right hip and nondominant wrist showed 90% accuracy for recognition of sedentary behavior among preschoolers. Authors used Radom Forest and support vector machine classifiers. 

Rawassizadeh et al. [8] highlighted the research challenges and opportunities associated with smartwatches. Authors identified the battery life, cost and optimizing hardware resources as the major challenges for smartwatch acceptance in the market. They highlighted the potential of smartwatches to persuade users towards a more active lifestyle. However, the authors indicated that algorithms that focus on energy-efficient activity recognition need further development. Mortazavi et al. [9] examined exergaming as a tool to fight with sedentary behavior. These authors developed a wearable exergame SoccAR that involves high-intensity movements as the basis for control. Four Shimmer wireless inertial measurement units equipped with accelerometers and gyroscopes were attached to the volunteer’s wrists and legs. The game was displayed on the head-worn display—the Epson Moverio BT-100. The emerging approach is also the use of smartphones equipped with accelerometers. Fahim et al. [10] showed that smartphone could be used to monitor sedentary behavior. Authors developed smartphone application “Alert Me” that notified the user in order to avoid prolonged sitting based on the analysis of tri-axial accelerometer data. Qian et al. [11] proposed A Rhythm Analysis-Based Model of sedentary behavior based on smartphones-sensed user activity logs that may be generated by smartphones applications to achieve better behavior change outcomes. Shin et al. [12] noticed that many types of research did not consider device orientation what makes it difficult to accurately classify sedentary behavior. The device was mostly fixed to arm, wrist, hip, thigh, and shank. They proposed a method to recognize sedentary activity with the acceleration data rotated by quaternion to face this challenge. The emerging approach is also the use of smartphones equipped with accelerometers and information about micro-context. Fahim et al. in [13] investigated a user-centric smartphone-based approach to recognize the context of sedentary behavior based on the onboard accelerometers and audio sensors of the smartphone. Authors showed that developed application is capable of reducing sedentary behavior. 

Qian et al. [14] explored the contexts of information that can be sensed by subjects’ smartphones can be used to predict their future sedentary behavior. They analyzed 49 college student data and using logistic regression were able to predict that subject will be sedentary in the next hour with the recall of 87.7%.

Ma et al. [15] developed a smart cushion and system that monitor sitting postures. It features micro controller unit, pressure sensor, Bluetooth communication unit, vibration motor, and power supply circuit. Eight persons aged 60–65 years were recruited to the study. Participants were asked to perform common activities on the wheelchair for about 2 h. Results showed that the overall average recognition accuracy was 98% based on specific decision tree implementation. Authors concluded that investigation the integration of physiological sensors data is desired in the future research. Previous works highlighted the importance of investigating novel wearable computing platforms, sensor modalities and algorithms for resource-constrained environments in order to monitor human behavioral pattern for healthcare application. 

This study addresses the need to encourage especially older people to be more physically active. The main objective of this study was to develop the method of automatic recognition of sedentary behavior related to cardiovascular risk based on quantitative measurement of physical activity. The solution is based on the designed smart shirt equipped with a processor, wearable sensors, power supply and telemedical interface. 

The novelty of the proposed method is twofold. First, it uses sensors with two different modalities that are fused into a feature vector and passed to classification models to estimate sedentary behavior. Second, it uses the prototype of smart shirt integrated with the telemedical interface that integrates the measurement chain and the computational part.

This approach may be especially useful for hospital patients who need to be constantly monitored outside the ICUs or older adults who are unable to deal with sophisticated medical devices and need to be monitored remotely. The hypothesis of the study is that the signals collected by smart shirt contain information to recognize sedentary behavior during activities of daily living and can be transmitted to the telemedical service provider for storage and further analysis.

However, there was a gap in the literature concerning the method for multimodal inactivity recognition system optimized to wearable resource-constrained computing platforms. The proposed method is based on a personalized approach to medicine and prevention. The focus shifts from the treatment of the disease to its prevention based on updated continuously pattern of patient behavior, and its interaction with the environment regardless of location. The rest of the paper is organized as follows: related works and market demand in Section 2. Section 3 describes the material and methods. Results are investigated in Section 4. Section 5 discusses the results. Finally, conclusions and future work are presented in Section 6.

## 2. Related Works and Market Demand

The latest developments in wearable computing technology and biosensor enable the recording of movement and biosignals in different environments. The main advantage of these technologies is their ability to record patient activity in a free-living environment continuously.

The most widely used method of continuous patient cardiac monitoring is the use of a portable device for the monitoring of the electrical activity of the cardiovascular system (Holter ECG), which allows continuous recording of the electrocardiogram signal for supporting the diagnosis of atrial conduction dysfunction syndromes and therapy monitoring [16]. 

Report “Patient Monitoring Devices Market—Global Industry Analysis, Size, Share, Growth, Trends and Forecast 2014–2020” carried out by Transparency Market Research estimated that the global patient monitoring market size is valued at 31.4 billion USD and will grow at 14.2% annually by 2020 [17]. The report titled “Global Markets for Telemedicine Technologies” showed that the global telemedicine market reached 16.3 billion USD in 2013 and 19.2 billion USD in 2014, and it is forecasted to reach approximately 43.4 billion USD in 2019 with an average annual increase 17.7% with the home telecare as the fastest growing market sector. Its value amounted to almost USD 8.2 billion in 2014, and it is projected to USD 23.9 billion in 2019 (average annual 24% growth during the forecast period) [18]. The global fitness tracker market size was valued at 17.9 billion USD in 2016 according to Applied Market Research Report and is expected to reach $62. 1 billion USD by 2023 (average annual 19.6% growth during the forecast period) [19]. The growing fitness tracker market proves the high demand for easy to use health tracking devices, which do not limit the everyday activities. They track the number of steps, distance, calories and sleep quality. However, they are often designed as gadgets and not clinically validated medical devices, and they are not intended for supporting the diagnosis or disease prevention. 

The concept of using the smart shirt to acquire physiological signals is not new. With the advances in wearable technologies, smart shirts have been introduced to the consumer market. The Hexoskin smart shirt uses body-worn sensors to capture ECG data (one channel, 256 Hz), heart and breathing rate, tidal volume, minute ventilation, acceleration signal (three channels, 64 Hz) and hip motion intensity (HMI) during different body positions including lying, sitting and standing [20]. Nuubo is an elastic vest that offers an ECG monitoring system that enables physicians to diagnose arrhythmias. It was developed in Spain. ECG electrodes are printed in 3D into the wearable textile. The Nuubo monitoring system enables five days autonomy battery operation and up to 30 days recording of 3 axis acceleration and arrhythmias. Fabregat-Andres et al. used the Nuubo’s technology for cardiac screening in soccer players and acquired the accuracy of single-lead electrocardiographic recordings during exercise testing in the field [21]. The Equivital EQ02+ LifeMonitor provides multi-parameter monitoring capabilities, including ECG, Respiratory and 3D accelerometer. It consists of two components: the Sensor Electronics Module and the Sensor Belt, that can be worn 24 h/day and is machine washable. It was developed in the United Kingdom. Akintola et al. concluded in his study that although the Equivital EQ02 can accurately measure ECG and HRV, its accuracy and precision is highly dependent on artifact content [22]. The Zephyr BioHarness is wearable devices that is attached to the chest and can perform real-time physiological monitoring of heart rate, breathing rate, and posture. It features 8-h run-time on a single chargé and remote real-time viewing of data within a two-mile radius with the portable wireless transmitter. Kim et al. [23] tested BioHarness to determine the accuracy of heart rate (HR) and respiratory rate (RR) measurements during two exercise protocols in conjunction with either a laboratory metabolic cart (Vmax) or a previously validated portable metabolic system. It was found that correlation coefficients between the methods were low for HR but moderate to highly correlated (0.49–0.99) for RR [23]. The Corscience CorBELT is a chest strap which continuously measures and analyzes a 1-channel ECG. It can be used as ECG monitor or an event recorder. The CorBELT analyzes the ECG and, if an arrhythmia occurs, records a 2-min ECG (1 min before and 1 min after the arrhythmia). It can store about 20 min of recordings or 10 events. The recorded measurements are transmitted using Bluetooth to PDA or cell phone. Steven Wieland et al. created a system for the recognition of atrial fibrillation and calculation of risk level based on the chest strap CorBELT [24]. A survey on human activity recognition (HAR) has found 28 prototypes of systems which were evaluated in terms of recognition performance, energy consumption, obtrusiveness, and flexibility [25,26,27,28]. The accuracy of HAR varied from 56% to 94% and was based mostly on acceleration data. 

Prior work has proposed various knowledge extraction techniques from the wearable dataset. Rawassizadeh et al. [29] investigated scalable algorithms that analyze multivariate temporal data in order to identify human behavioral patterns. The developed solution is based on a temporal granularity method which assumes that our daily behaviors occur in time intervals. It also employs a combination of different sensors instead of specific sensors to reduce uncertainty by using only sensors that are available. The developed algorithms can be implemented in smartwatches and do not require cloud processing. Nath [30] proposed the acquisition context engine (ACE) based on context caching and context rules for continuous sensing of the user’s context in a mobile device. He confirmed that ACE could reduce the overall energy consumption. Wang et al. [31] showed the real-time drug use detection method based on the outlier analysis and slide window technique using data obtained from wearable biosensors. A recent review [32] of the latest methods used to analyze data from wearable sensors reveals that the data mining technique is dependent on the mining task to be performed. Support vector machine, neural networks, and decision tree techniques applied for general health care problems usually give satisfactory results. However, methods including neural networks, Gaussian mixture model and frequency analysis are not efficient in real-time health monitoring systems because of their computational complexity. Decision tree, rule-based and statistical techniques are often used for the real-time data processing. The critical factor to consider in investigating data mining for healthcare is adequate data labeling. A recent survey [33] of data mining systems using wearable devices reveals that privacy and security-related concerns are arising. Authors concluded that data mining algorithms should be optimized for cost and computation reduction. Lightweight algorithms should be computed by the wearable device and prioritized over complex algorithms, that need to be computed in the cloud environment.

Our previous studies focused on the investigation of various sensors and algorithms for application in wearable healthcare monitoring systems. In [34] we described a multimodal system for seamless surveillance of the elderly in their living environment. We also investigated the integration of wearable ECG and accelerometer sensors for human telemonitoring [35]. Although significant progress has been made in human activity monitoring, there are still some challenges that should be addressed to accelerate the development and adoption of wearable telemedical services. The major challenges are a multimodal signal acquisition, resource-constrained computing environment, difficulty to operate, power management, device size and safety, limitation of performing activities of daily living activities during measurements and authorized access to health data. Anthroposociological changes will influence the development of the ubiquitous e-health services. We notice the increase in trust and acceptance of the society for the use of telemedical services. The development of new methods, aggregation models, and medical data analysis can affect the quality of telemedical health care services. 

## 3. Materials and Methods

### 3.1. Study Design

The intervention study was performed from 2016 to 2017 among adult volunteers. The inclusion criteria were written informed consent. Five adults (three females and two males) participated in this study (40 ± 10 years old, 170 ± 12 cm and 78 ± 20 kg). In contrast to previous studies that used different types of accelerometer sensors for activity recognition mounted of the wrist, belts, legs, or arms participants were asked to wear designed and developed a prototype of the smart shirt integrated with not only accelerometer sensors but also with a pulse sensor and ambient sensors. A set of experiments was designed to test the feasibility of the developed acquisition process and the accuracy of inactivity classification algorithm, ensuring adequate evaluation of the developed method. The measurements were performed in the home-based environment to simulate the free-living environment with each subject performing a set of 12 directed tasks according to guided voice commands from a computer application. The following tasks were selected:

- Ambulatory activities: lying, sitting, standing still, walking.
- Daily activities: working at the PC.
- The short physical performance battery (SPPB) test tasks: sit-to-stand, feet together standing, semi-tandem standing, full tandem standing, 4-m walk.

**Figure 1:**
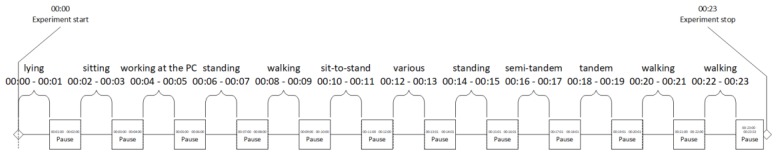
Task sequence and duration.

We included tasks from the short physical performance battery (SPPB) test that is considered as a not inactive state but can be safely performed by older people. Each task lasted 60 s and was proceeded by a 60-s break for preparing for it. Full recording for one subject consisted of 28,800 samples and lasted 23 min. Each window was automatically labeled. Figure 1 exemplifies the task sequence and duration. 

### 3.2. System Architecture

The primary principle in the design of the wearable health monitoring system is to achieve a real-time classification of subjects’ inactivity using smart shirt integrated with the processor, multiple sensors, LEDs, power supply and wireless radio during activities of daily living. The developed shirt prototype is designed to be easy to use and operate especially for older or disabled people in the home-based environment or a clinical setting. 

The system consists of three types of nodes which communicate in three following overlapping networks: Personal Area Network (PAN) that is created dynamically in the proximity of the user, Local Area Network (LAN) and the Internet. 

**Figure 2:**
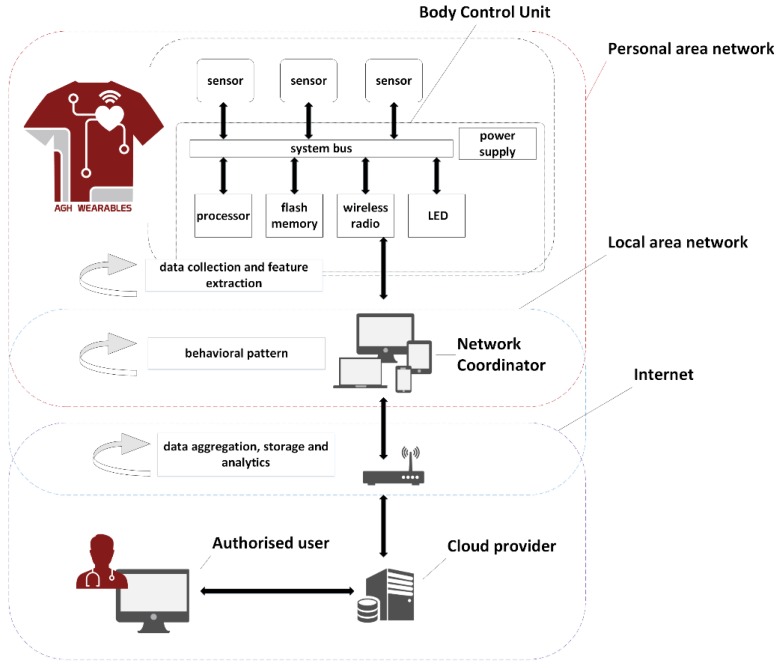
System architecture.

Body control unit (BCU) is sewn on the shirt. It creates the PAN network and waits for incoming connections. If the network coordinator (NC) connects to the BCU, the connection is established. BCU collects, filter and encapsulates data as well as extracts features and communicate with the PAN network with network coordinator (NC). NC acquire and analyze data packets from BCU and transmits results to the cloud provider (CP) via available Local Area Network (or Wireless Local Area Network) interface. Cloud provider (CP) performs data aggregation, storage, and further analytics as well as provides remote data access interface for authorized users. The system can be configured to operate only within the Local Area Network to limit data exchange with the external service provider. The system architecture is shown in Figure 2. 

### 3.3. Instrumentation

**Figure 3:**
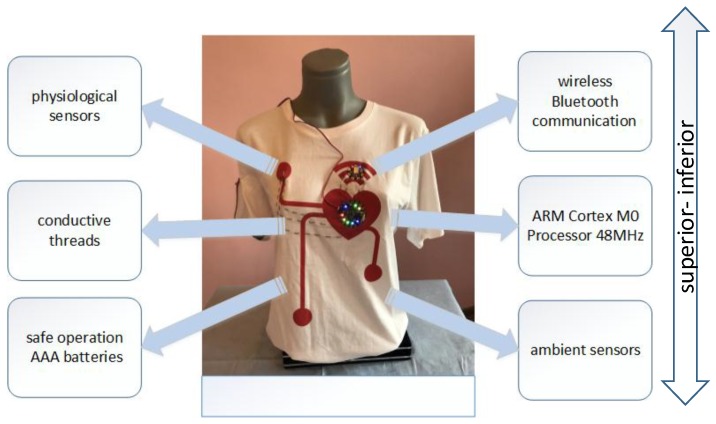
The system prototype.

The main idea was to develop the prototype of wearable health monitoring system that can acquire selected signals in a free-living environment. To face this challenge the components were integrated into the prototype of the smart shirt. The design of the system was based on the off-the-shelves open-hardware components. The wearable health monitoring system consists of the low-power 48 MHz ARM Cortex M0 processor that features various peripherals which facilitate communication with different sensors and design of the multisensor system. The processor supports software-selectable sleep modes, up to six Serial Communication Modules (SERCOM) which can be configured to act as UART, SPI, I^2^C and up to twenty-channel 350 ksps 12-bit Analog-to-Digital Converter (ADC) with programmable gain. We used the ultra-low-power high-performance three-axis LIS3DH linear accelerometer with digital I2C/SPI serial interface standard output. The device is capable of measuring accelerations with output data rates from 1 Hz to 5.3 kHz and may be configured to generate interrupt signals. It has an integrated 32-level first-in, first out (FIFO) buffer allowing the user to store data to limit intervention by the host processor. For the ambient light sensor, we used ALS-PT19-315C. The peak sensitivity of this sensor is around 640 nm. The temperature was measured using NCP15XH103 chip that features high accuracy in resistance and high stability in the environment. We used an optical heart-rate sensor based on open source hardware Pulse Sensor developed by Murphy and Gitman [36], that was previously validated by the community. Pulse Sensor is based on the Avago light sensor (peak sensitivity for this sensor is 565 nm) and the green super bright LED from Kingbright. The hardware uses a filter and amplifier to increase the amplitude of the pulse wave and normalize the signal around a reference point. Pulse sensor was connected to the microcontroller using conductive fabric. The device is powered by three AAA rechargeable batteries to ensure safe operation with close contact with the body and potential issues during activities of daily living. Wireless communication is facilitated by the MDBT40 Bluetooth Low Energy (BLE) module designed based on Nordic nRF51822 SoC solution. It features ARM Cortex M0 32 bit processor, the dual transmission mode of BLE and RF 2.4G, −93 dbm sensitivity in Bluetooth low energy mode and low power requirements. It also provides full coverage of BLE software stack including Heart Rate Profile, Health Thermometer Profile, and Blood Pressure Profile. The conductive thread was used to connect system components. The system prototype is shown in Figure 3. 

### 3.4. Feature Extraction and Classification Algorithm

The sedentary behavior recognition process described in this paper was divided into the following steps. The developed method was based on supervised machine learning algorithms. The major challenge was to select the sensors’ sampling rate that compromises the size of the data stream, computing power and to acquire sufficient information to describe the performed activity or health state (abnormal state). The various windows size and sampling frequency were analyzed to face the limits of wearable computing platform. Activity states are recognized in a prolonged 60 s time window basis to obtain sufficient descriptive information and to support the implementation of SPPB Protocol. Signals sampling frequency was set to 40 Hz what is higher than state of the art solutions such as activPAL (20 Hz) or Active style Pro (32 Hz). 

The proposed approach is based on applying the following feature extraction method to each time window and obtaining quantitative measures. The estimation of heart rate is based on the analysis of the acquired pulse wave vector. The signal peaks above a threshold value of 540 (~1.74 V) and with a minimum distance of 19 samples are marked and used for calculating the heart rate. During our experiments we investigated the following time domain features of accelerometer and light intensity measurements including sum, mean, standard deviation, variance and root mean square. A vector of the dominant frequency for each axis was selected from frequency domain features of accelerometer measurements. We examined heart rate variability measures such as the standard deviation of beat-to-beat intervals and the square root of the mean of the squares of the successive differences between neighboring beat-to-beat intervals. Acceleration signals were filtered using sixth order highpass Butterworth filter with a cutoff frequency of 0.25 Hz and a window size of 10 samples.

Finally, we selected the following features: relative intensity $\left(I_{R}\right)$ (defined in Equation (1)) and the variance of the acceleration signal (A) of the *x*-axis (superior-inferior) after filtration (defined in Equation (2)):

(1) $$\;I_{R}=\frac{\omega }{\varphi }\times 100\%\;$$

where ω is heart rate, φ is estimated maximum heart rate.

(2) $$V=\frac{1}{N-1}\sum_{i=1}^{N}{\left|A_{i}-\mu \right|}^{2}$$

where μ is the mean of *A*, $\mu =\frac{1}{N-1}\sum_{i=1}^{N}A_{i}$.

In the second step, the obtained two-dimensional feature vector that passed through to the classification model, which tried to classify the appropriate class. The first class of activities was chosen as sedentary behavior: lying, sitting, working on the PC. The second class activity was the remaining activities. The data acquired during experiments were labeled manually with the appropriate class and saved in a database. We investigated the following classification models: Binary decision trees, Discriminant Analysis model, Naive Bayes (Gaussian), k-nearest neighbors classification (k = 5, distance = Euclidean), Support vector machines for binary classification and artificial neural networks with the following topology: one neuron has been placed on the output layer, 12 neurons on the hidden layer and two neurons in the input layer. We validated the classification models using 10-fold cross-validation.

## 4. Results

**Figure 4:**
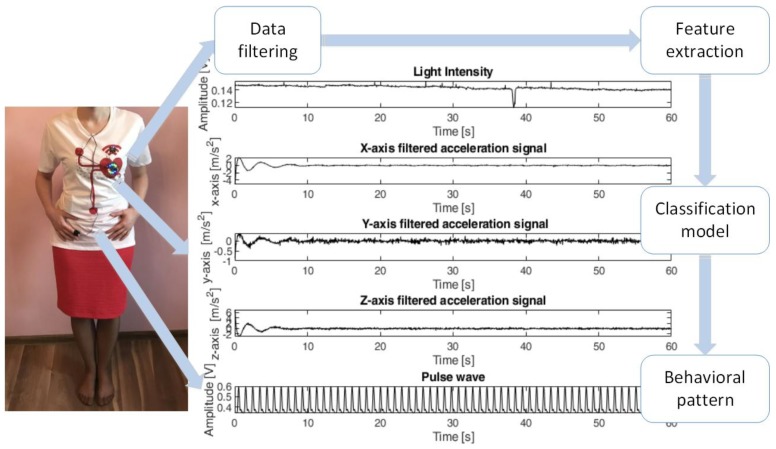
The diagram displays standing volunteer during experiment protocol and the plot of light intensity, acceleration, and pulse wave signals as well as output from classification model.

The results of the experiment confirmed the initial hypothesis about the feasibility of a developed method to acquire and analyze multi-sensor signals during experimental protocol. Figure 4 displays standing volunteer during experiment protocol and the plot of light intensity, acceleration, and pulse wave signals as well as output from classification model. 

**Figure 5:**
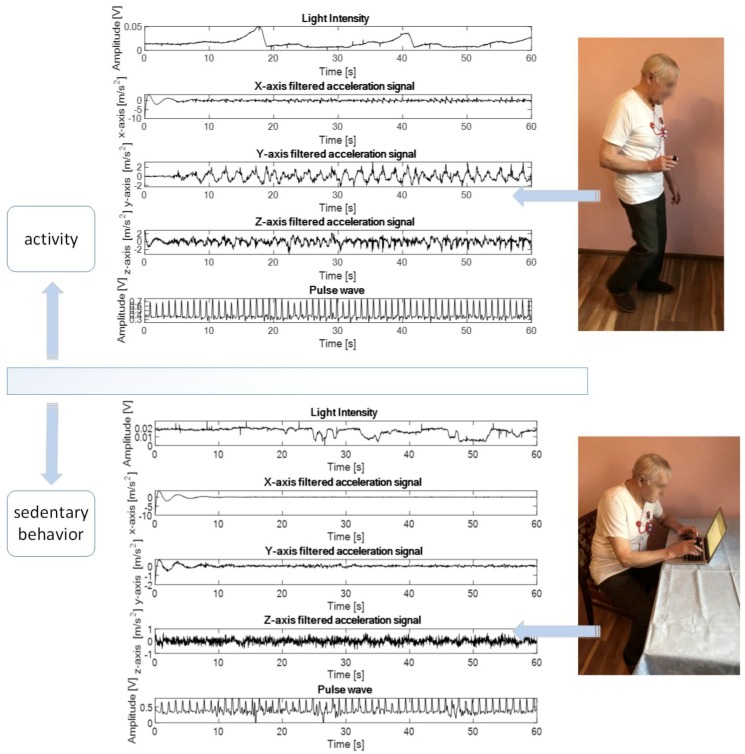
The diagram displays volunteer while walking and sitting and the plot of light intensity, acceleration, and pulse wave sensor data as well as output from classification model.

Additionally, Figure 5 displays volunteer while walking and sitting and the plot of light intensity, acceleration, and pulse wave sensor data as well as output from classification model.

**Figure 6:**
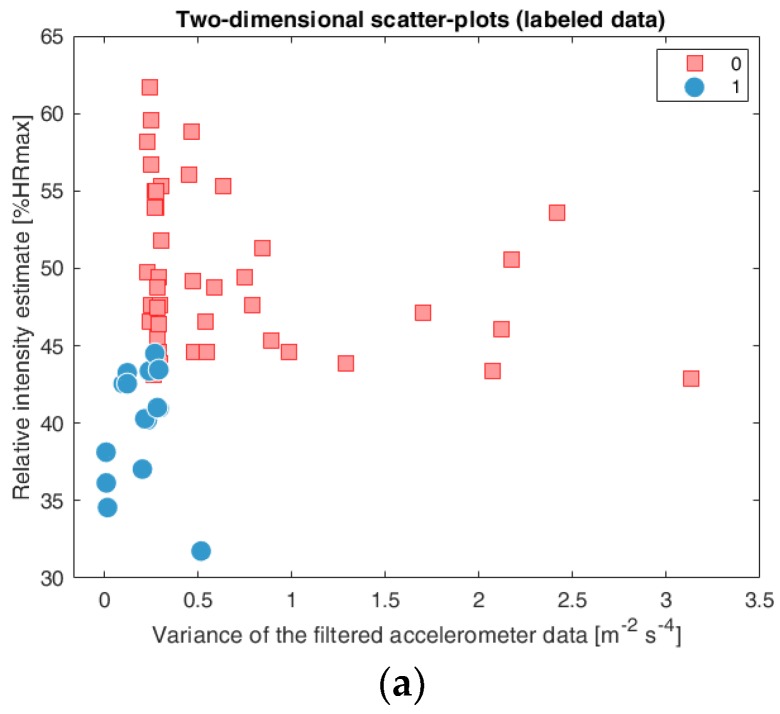

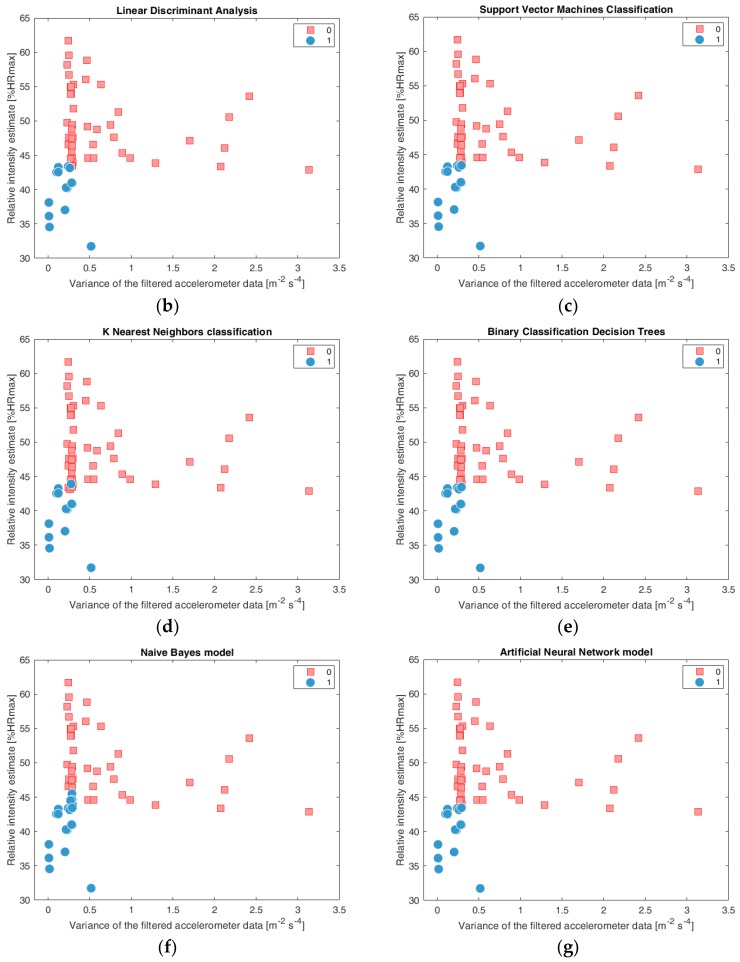
The diagram of transformation of wearable sensor signal into feature space by a machine learning approach: two-dimensional scatter-plots (labeled data) (**a**), Linear Discriminant Analysis (**b**), Support Vector Machines (**c**), K Nearest Neighbors (**d**), Binary Decision Trees (**e**), Naive Bayes (**f**), Artificial Neural Network (**g**).

In this experiment, we investigated six different classification models to measure the performance of the proposed method. The developed methods were used to transform sensor signals into a two-dimensional feature vector. Figure 6a–g display two-dimensional feature vectors scatter-plot consisted of the variance of the relative intensity estimate and filtered accelerometer data. Figure 6a displays labeled data for the reference purpose. The blue circles represent inactivity while the red squares represent activity. Figure 6b–g display the results of investigated models classification (b) Linear Discriminant Analysis, (c) Binary Decision Trees, (d) K Nearest Neighbors classification, (e) Support Vector Machines Classification, (f) Naive Bayes, (g) Artificial Neural Network).

The results of the assessment of the proposed method are summarized in Table 1. The following metrics were defied in Equations (3)–(7).

(3) $$\mathrm{Accuracy}=\frac{TP+TN\;}{N}\;$$

where TP is true positive (the model correctly predicted the sedentary behavior pattern), TN is true negative (the model correctly predicted the non-sedentary behavior pattern), *N* is nua mber of all evaluated cases.

(4) $$\mathrm{Sensitivity}=\frac{TP}{TP+FN}\;$$

where FN is faa lse negative (the model incorrectly predicted the non-sedentary behavior pattern).

(5) $$\mathrm{Specificity}=\frac{TN}{TN+FP}$$

where TN is a true negative, FP is false positive (the model incorrectly predicts the sedentary behavior pattern).

(6) $$\mathrm{Precision}=\frac{TP}{TP+FP}$$

(7) $$\mathrm{F1-Score}=2\times \frac{\mathrm{Precision}*\mathrm{Specificity}}{\mathrm{Precision}+\mathrm{Specificity}}$$

The following three classifiers manifested the best performance in terms of accuracy: Support Vector Machines, Support Vector Machines, Artificial Neural Network. The Naive Bayes classifier manifested the worst performance in terms of accuracy. However, the average measurement results of metrics for all investigated model were: Accuracy = 95.00% ± 2.11%, Sensitivity = 91.11 ± 6.88%, Specificity= 96.29 ± 3.63%, Precision = 90.02 ± 7.37%, F1-Score = 90.18 ± 3.74%. 

## 5. Discussion

The experiment results confirmed the initial hypothesis about the feasibility of a developed method to acquire and analyze multi-sensor signals during activities of daily living based on the electronic shirt. The classification models were able to identify the sedentary behavior with an accuracy of 95.00% ± 2.11%.

Obtained results are satisfactory and similar to state-of-the-art results for human activity recognition (HAR). The overall accuracy of the state of the art algorithms for HAR varied from 84% to 97% [25,37,38,39,40]. However, the accuracy level comparison is challenging due to different sensor modalities, hardware architecture, measurement chain, sensor location and configuration as well as experimental protocol. Ambulation, fitness and daily activities were most commonly investigated. This is a general problem for all studies in this area and Sasai also confirmed it in his recent study [4]. Prior studies have been restricted to studies based on accelerometer mounted to the different body parts [41] or based on portable devices [38]. In addition, computationally complex algorithms unsuited to resource-restricted wearable platforms were used [42]. 

During experiments, it was observed that in some scenarios it is not possible to distinguish standing from sitting based only on accelerometer data. We decided to investigate other modality sensors to face this challenge, and we chose the pulse wave sensor. We observed that heart rate is significantly higher during non-sedentary behavior in comparison to sedentary behavior. 

This observation was confirmed by Koster et al. [6] who pointed out that wrist accelerometers underestimated the sedentary time and in some scenarios sitting time may not be distinguished from standing time based on accelerometer data. Furthermore, monitoring heart rate is desired in a clinical parameter in the telemedical setting. 

The initial hypothesis assumed that light signal might contain context information that can differentiate sedentary behavior. Although there were some promising results for periodic activities like sit-to-stand or walking, obtained results did not support this hypothesis. It will be investigated in the future research. 

Shin et al. [12] noticed that many types of research did not consider accelerometer device orientation what makes it difficult to classify sedentary behavior (i.e., vector magnitude feature) accurately. The device was mostly fixed to arm, wrist, hip, thigh and shank. They also noted that sitting and standing are difficult do recognize because there is no much difference in acceleration.

The proposed method faces the challenges discussed in this paper because it employs multimodal sensors and provides some advantages over portable devices in terms of wearability, ease of use and flexibility. Furthermore, the accelerometer sensor is sewn to the center of the shirt while the physiological sensor is attached to the conductive thread (the right-upper part of the shirt) using elastic cable in order to acquire pulse wave signal from three different location depending on the desired configuration including finger, arm or ear. 

Proposed feature vector was designed to face the limitation of restricted resource environment and consisted of low computational complexity parameters that are individually calculated and includes relative intensity parameter and filtered x-axis motion variation.

According to Centers for Disease Control and Prevention (Disease Control and Prevention, LINK) relative intensity is defined as the level of effort to perform an activity. Individuals who are less fit require a higher level of effort than fitter people to perform the same activity. It is determined as an individual ratio of person’s measured and estimated heart rate (%HRmax), which is 220—age. This idea laid the foundation for the developed method. Relative light intensity level 50–63% of HRmax (or 1.1–2.9 MET) takes place while walking < 4.7 km/h or light housework work while relative moderate intensity level 64–76% of HRmax (or 3–5.9 MET) takes place during walking briskly (4.8–6.5 km/h), vacuuming or gardening. Based on experimental results the relative intensity during sedentary behavior among all subjects ranged from 31% to 44% of HRmax while other tasks varied from 45% to 61% HRmax. According to [2] doctors should evaluate the physical activity in every patient. They should warn against inactivity and recommend adding physical activity to daily life. However, the automatic monitoring of inactivity is a challenge, especially among older adults who are unable to use existing medical devices on a daily basis.

During experiments we investigated the power consumption. The prototype was connected to 9 V Energizer 175 mA, and it operated for 156 min. It is estimated that using four AAA 1000 mA batteries, it can last up to 3 days. 

One of the major contributions of this research is the design of smart shirt-based multimodal sedentary behavior monitoring system and its validation during experimental protocol which was designed to be safe for the elderly and was based on clinically validated short physical performance battery (SPPB) test tasks that is the gold standard for elderly clinical examination. 

To the best of our knowledge, this paper is the first to study sedentary behavior using smart shirt-based multimodal sedentary behavior monitoring system, six machine learnings models and purposely design experiment protocol suitable for older adults as well as the design of telemedical service. Continuous tracking of quantitative health-related parameters, as well as their context, can contribute to better understanding of patients’ health state and may support the early detection of diseases as well as prevention and treatment. In addition, estimating the behavior pattern can support making better health decision before the symptoms of diseases (CVDs diseases) appear.

The way of diagnosis and treatment is also changing. ICT tools and new medical technologies are playing an increasingly important role in this process. The contemporary paradigm of evidence-based medicine evolves towards an approach based on personalized medicine and prevention. The concentration shifts from the treatment of the disease to its prevention based on constantly updated health data, its pattern of behavior and its interactions with the environment.

With the increased role of medical devices in the diagnosis and treatment process, a key aspect in the assessment of devices is to ensure that the requirements for a specific application are met. It is essential to take into account functional requirements, risk analysis, parameter and safety requirements in accordance with the product application, legal requirements and regulations (e.g., European Medical Devices Directive 93/42/EEC, FDA). Meeting these requirements can be confirmed by a certification audit performed by an independent accredited organization. Therefore, the development of dedicated wearable medical devices seems to be the most promising and having an advantage over general purpose solutions. 

The study has some limitations. We selected the most common activities that are performed by older adults at home or in the hospital setting including lying, sitting, standing and walking. Other complex activities should also be evaluated in future studies. Affective states including stress should be investigated because are associated with a high heart rate. Our previous experiment showed that wearable sensors could also be used to recognize affective states. We have also proved the influence of emotions on visual acuity, the feasibility of wearable eye-tracking-based assessment of emotional state [43]. For patients taking medications, it is important to consider the possibility of modifying the heart rate response.

The systems’ dependence on external telecommunication service provider is a significant threat and limitation for the proper functioning of the service. In case of materializing the risk regarding the lack of access to the network, it is not possible to exchange data with the central server. As a risk-minimizing measure, data recording on the device and signaling with the use of LEDs can be used as the communication interface with the user or personnel. Rawassizadeh et al. [44] recommended prioritizing on-device analysis over uploading the data to the cloud service due to threats to privacy, advances in hardware capabilities and cyber-attacks. Supervision over the storage of medical data is an essential aspect for the stakeholders, therefore, apart from cloud solutions, it is important to provide the possibility of using local hospital networks. 

## 6. Conclusions

In conclusion, a sedentary behavior can be recognized from the fusion of the data from smart shirt, individual factors, and machine learning algorithms. The findings demonstrated that the proposed method could be used to quantitative measure sedentary behavior without limiting daily activities. Experimental results suggested that high accuracy can be obtained by estimating the sedentary behavior pattern using machine learning approach. In addition, personalized shirt-based health telemonitoring system can provide clinically significant information about subjects’ behavior to health providers or authorized users. This could potentially be used for early diagnosis, prevention, and treatment as well as could motivate to more active lifestyle, especially for frail older adults.

## 7. Patents

P.418874 The method of signal acquisition, sensor sticker and control-measurement system.

## Figures and Tables

**Table 1:** The assessment of the proposed method.

No	Method	Accuracy	Sensitivity	Specificity	Precision	F1-Score
1	Linear Discriminant Analysis	95.00	86.67	97.78	92.86	89.66
2	Support Vector Machines	96.67	93.33	97.78	93.33	93.33
3	K Nearest Neighbors	93.33	80.00	97.78	92.31	85.71
4	Binary Decision Trees	96.67	93.33	97.78	93.33	93.33
5	Naive Bayes	91.67	100.00	88.89	75.00	85.71
6	Artificial Neural Network	96.67	93.33	97.78	93.33	93.33
**Average**		**95.00**	**91.11**	**96.29**	**90.02**	**90.18**
